# The current condition of the workers’ general health examination in South Korea: a retrospective study

**DOI:** 10.1186/s40557-017-0157-0

**Published:** 2017-03-07

**Authors:** Young Joong Kang, Jun-Pyo Myong, Huisu Eom, Bowha Choi, Jong Heon Park, Eun-A L Kim

**Affiliations:** Occupational Safety and Health Research Institute, Korea Occupational Safety and Health Agency, Ulsan, Republic of Korea

**Keywords:** National health screening system, Worker's general health examination, National general examination, History of periodic health examination, Participation rate, Hypertension prevalence, Diabetes prevalence, Small enterprise, Health inequality

## Abstract

**Background:**

Business owners in the Republic of Korea must take part in the workers’ general health examination. However, there have been few formal analyses of the uptake of this examination by employees. In the present study, we examined the rates of participation in medical examinations according to age group, health insurance type, and enterprise size, and then compared these results with those of the national general health screening. Furthermore, we determined the distribution of patients with abnormal results for diabetes and hypertension, and outlined the significance and history of domestic health examinations.

**Methods:**

We started by comparing participation rates extracted from the among health examination data of the National Health Insurance Service from 2006–2013 by sex, age, insurance type, and enterprise size of workplace health insurance beneficiaries (i.e., those whose insurance is provided by their workplace). In addition, we analyzed the prevalence rates of abnormal results for hypertension and diabetes, and explored the history and significance of health examinations in the Republic of Korea.

**Results:**

The overall participation rate in the primary health examination in 2006 was 56%, and this increased to 72% in 2013. However, the rates of the secondary screening did not increase much. Among workplace policyholders (i.e., those whose insurance is provided by their workplace), the participation rates of workers in enterprises with less than 50 employees were lower than were those in enterprises with 50 or more employees. Notably, the rates and odds ratios of patients with abnormal results for diabetes and hypertension were relatively high, particularly among those working in smaller enterprises.

**Conclusions:**

Although the workers’ general health examination has been replaced with the national general health screening, it remains necessary to ensure uniform health management services among all workers in the Republic of Korea. This can, in turn, promote occupational health and improve working conditions throughout the Republic of Korea.

## Background

In the Republic of Korea, business owners are obligated to take part in the workers’ general health examination (WGHE), in accordance with the Industrial Safety and Health Act, as part of efforts to protect workers’ health. The WGHE is differentiated from the workers’ special health examination (WSHE) in that the latter comprises 178 items for workers who are regularly exposed to 177 hazardous substances and various physical environments specified by law, as well as night shift workers, whereas the former is administered to regular workers only.

However, according to an existing law, the WGHE can be substituted with the national general health screening, which is covered by National Health Insurance Service [1]; as such, unlike the WGHE and WSHE, employers do not need to pay for provision of the national general health screening. Furthermore, it is generally recognized that accessibility of the results of the WGHE is somewhat lower than is that for the results of the WSHE, as the latter results must be reported by the employer to the Korea Occupational Safety and Health Agency database, given that it is applicable only to workers with clearer risks and who are working more hazardous environments. For this reason, there has been little research interest in the WGHE in the field of occupational medicine.

As with other Korean health screening systems, there have been a number of studies concerning the national general health examination (NGHE) of the Korea's National Health Insurance Service (NHIS). However, most of them focused on the effects of this screening rather than on industrial health and workers’ health management. Furthermore, very few of these studies directly analyzed the official dataset provided by the NHIS [2, 3]. Prior studies on the evaluation of national health screening programs in Korea used simulation techniques [4, 5]. Accordingly, there has been no analysis of the actual conditions or effect of the WGHE.

The role of the WGHE, according to the Industrial Safety and Health Act, is currently played by the NGHE. Previous studies have grasped the actual conditions and effects of health examinations utilizing the national general health examination conducted by the NHIS for workplace policyholders and regional policyholders. However, although the NGHE has roughly the same items as the WGHE, the WGHE is used not only as a form of health screening nationwide but also as a means of health surveillance, which is one of the 11 basic duties that business owners have to protect concerning workers’ physical and mental health according to the Occupational Health Services Convention (C161) of the International Labor Organization [6]. Thus, we focused on analyzing and investigating aspects of this health examination in the terms of occupational medicine. More specifically, we determined the participation rates in health screenings among workers using data from the National Health Insurance Service and the prevalence of abnormal results for diabetes and hypertension, which can precede severe cardio- and cerebrovascular diseases (CCVDs). CCVDs are a major cause of death and workforce loss among workers and individuals in the general population. The prevalence of abnormal results for diabetes and hypertension were stratified by workers’ insurance type and enterprise size. Furthermore, we wanted to examine the differences in the odds of having major two chronic diseases—diabetes and hypertension—according to workers’ enterprise size, with a focus on whether having a small workplace can affect prevalence of diabetes and hypertension. Accordingly, we determined the odd ratios of diabetes and hypertension among workplace policyholders in their 40s and 50s by enterprise size.

In addition, we have attempted to discuss the Korean health screening system as a whole by reflecting on the history of health screening. We specifically consider when the concept of periodic health screening began and when Korea founded and structured the modern national general health examination system and workers’ health examination system. Furthermore, we describe how the WGHE came to be substituted with the NGHE, and other major institutional shifts in the health care system in the Republic of Korea. By doing so, we hope to contextualize the current state of health screening and look ahead to improving the occupational health care system.

## Methods

### Workers’ general health examination (WGHE)

The Ministry of Employment and Labor obligates business owners to provide workers with health examination service. Business owners arrange the WGHE for office workers and non-office workers not eligible for the WSHE (i.e., those exposed regularly to 177 hazardous substances and physical factors specified by the Industrial Safety and Health Act or night shift workers). As noted above, the WGHE may be substituted with the NGHE provided by the NHIS.

### National general health examination (NGHE)

The NGHE is a nationwide health screening that does not merely focus on individuals with pathologic symptoms and signs; instead, it targets all health insurance beneficiaries. This health screening comprises an interview by physicians, blood lab data, urine analysis, chest radiography, and measurements of body weight, height, eyesight, and hearing ability. The NGHE includes all of the WGHE items except the urine dipstick test. Fasting glucose level and blood pressure tests, detailed questions to patients, physical measurements, eyesight and hearing tests, and chest radiographies are all included. The blood tests examine hemoglobin, total cholesterol, high-density lipoprotein cholesterol, triglycerides, and the liver enzyme test (including aspartate and alanine transaminase (AST/ALT), gamma-glutamyl transferase, serum creatinine etc.). The amount of protein in the urine is also tested. Cognitive dysfunction tests are also selectively performed for elderly people.

The National Health Insurance Service actually provides a variety of major health screening services aside from the NGHE, such as health screening for lifetime transition periods, dental health screening, cancer screenings, and infant health screenings. The NGHE service is based on health examination criteria specifically for workplace and regional policyholders. Workplace policyholders are individuals whose insurance is provided by their workplace, and include office workers (i.e., individuals working in general affairs, personnel management, administration, sales, design, etc.; in other words, workers who are not manual laborers), non-office workers (employees other than office workers), and their dependents (aged 40 or older). Regional policyholders include “owner-operators” and self-employed workers, whose insurance is provided based on their own income, and their household members (aged 40 or older). Workplace policyholders who are non-office workers receive annual health examination services, whereas regional policyholders and workplace policyholders who are office workers (along with their dependents aged 40 or older) receive health examination opportunities on a biennial basis [7].

A secondary health examination is conducted for individuals suspected of having hypertension or diabetes as well as individuals at high risk for cognitive dysfunction after the NGHE (i.e., the primary examination). This secondary examination involves more detailed questions, blood pressure measurements, and blood tests to specify the abnormal results and determine a diagnosis (e.g., of hypertension or diabetes). Individuals who were found, in either the primary or secondary examination, to require further follow-up with a physician based on suspected hypertension or diabetes were classified as “examinees with abnormal results.” These two conditions were focused on because one of the main purposes of health examinations in the Republic of Korea is prevention of CCVDs and other chronic diseases.

This study dataset was derived from the NHIS, which contains four databases: insurance qualification, medical treatment charges, national health screening program results, and medical institution data. We obtained data from the insurance qualification and NGHE databases. More specifically, information on age, sex, type of insurance, and enterprise size were extracted from the insurance qualification database. The NGHE data obtained spanned 2006 to 2013; the eligible population during this period ranged from 14 to 17 million (Table 1).

**Table 1:** Participation rate in primary health examination

	2006			2007			2008			2009		
	TS^a^ (N)	P^b^ (n, %)		TS (N)	P (n, %)		TS (N)	P (n, %)		TS (N)	P (n, %)	
Overall participation rate	15,053,761	8,408,218	(56%)	14,358,709	8,419,532	(59%)	16,493,801	10,264,420	(62%)	16,277,239	10,537,866	(65%)
National health insurance type												
Workplace policyholder												
Non-public worker	6,138,566	4,756,163	(77%)	6,172,556	5,016,210	(81%)	7,411,510	5,953,473	(80%)	7,625,108	6,290,437	(82%)
Office worker	1,515,523	1,078,249	(71%)	1,072,442	839,517	(78%)	2782,176	2,044,152	(73%)	2,732,174	2,049,755	(75%)
Non-office worker	4,623,043	3,677,914	(80%)	5,100,114	4,176,693	(82%)	4,629,334	3,909,321	(84%)	4,892,934	4,240,682	(87%)
Public official	924,071	771,830	(84%)	278,547	213,526	(77%)	900,087	779,838	(87%)	240,178	195,910	(82%)
Dependents	2,915,863	1,383,217	(47%)	3,137,626	1,646,793	(52%)	3,380,998	1,831,665	(54%)	3,760,971	2,216,809	(59%)
Regional policyholder												
Householder	3,422,767	881,827	(26%)	3,230,823	920,406	(28%)	3,252,355	1,023,396	(31%)	3,131,656	1,113,517	(36%)
Household member	1,652,494	615,181	(37%)	1,539,157	622,597	(40%)	1,548,851	676,048	(44%)	1,519,326	721,193	(47%)
Enterprise size (no. of employees)												
≥ 300	3,919,021	2,825,502	(72%)	3,623,523	2,663,471	(74%)	4,210,329	3,195,498	(76%)	4,032,024	3,119,928	(77%)
50–299	2,518,852	1,905,700	(76%)	2,299,055	1,768,735	(77%)	2,803,298	2,235,709	(80%)	2,665,965	2,125,705	(80%)
< 50	3,540,576	2,179,986	(62%)	3,664,998	2,443,371	(67%)	4,678,882	3,133,715	(67%)	4,927,373	3,456,632	(70%)
Sex												
Male	8,345,124	4,858,065	(58%)	7,856,987	4,833,287	(62%)	8,992,965	5,763,063	(64%)	8,762,242	5,816,276	(66%)
Female	6,708,637	3,550,153	(53%)	6,501,722	3,586,245	(55%)	7,500,836	4,501,357	(60%)	7,514,997	4,721,590	(63%)
Age												
20s	1,549,890	1,188,441	(77%)	1,457,174	1,199,391	(82%)	1,755,391	1,413,162	(81%)	1,599,916	1,305,813	(82%)
30s	2,989,488	1,874,052	(63%)	2,652,807	1,764,649	(67%)	3,167,656	2,170,990	(69%)	2,883,761	2,014,012	(70%)
40s	4,556,426	2,252,394	(49%)	4,256,396	2,156,367	(51%)	4,796,802	2,677,379	(56%)	4,652,757	2,744,518	(59%)
50s	2,891,251	1,608,651	(56%)	2,826,586	1,620,394	(57%)	3,303,862	2,067,020	(63%)	3,443,763	2,235,227	(65%)
60s	1,795,284	984,003	(55%)	1,839,183	1,081,039	(59%)	2,016,084	1,260,900	(63%)	2,097,198	1,421,468	(68%)
70s	961,201	426,452	(44%)	1,002,204	504,698	(50%)	1,099,354	575,863	(52%)	1,202,184	692,595	(58%)
80s	259,015	56,875	(22%)	276,871	77,305	(28%)	302,444	82,816	(27%)	339,723	104,206	(31%)
90s	32,135	2,142	(7%)	31,629	3,063	(10%)	35,867	3,276	(9%)	39,774	4,676	(12%)
	2010			2011			2012			2013		
	TS (N)	P (n, %)		TS (N)	P (n, %)		TS (N)	P (n, %)		TS (N)	P (n, %)	
Overall participation rate	17,039,774	11,491,730	(67%)	16,333,464	11,739,774	(72%)	16,731,040	12,083,276	(72%)	16,911,464	12,108,885	(72%)
National health insurance type												
Workplace policyholder												
Non-public worker	7,707,049	6,414,938	(83%)	7,989,690	6,745,581	(84%)	8,018,933	6,578,513	(82%)	8,408,348	6,952,209	(83%)
Office worker	2,816,343	2,088,031	(74%)	3,061,514	2,302,668	(75%)	2,975,592	2,120,013	(71%)	3,042,002	2,091,641	(69%)
Non-office worker	4,890,706	4,326,907	(88%)	4,928,176	4,442,913	(90%)	5,043,341	4,458,500	(88%)	5,366,346	4,860,568	(91%)
Public official	689,527	606,349	(88%)	296,912	250,506	(84%)	638,023	569,189	(89%)	325,082	281,320	(87%)
Dependents	3,839,517	2,318,688	(60%)	3,684,603	2,444,912	(66%)	3,722,132	2,444,875	(66%)	3,860,906	2,563,906	(66%)
Regional policyholder												
Householder	3,264,589	1,350,573	(41%)	2,910,973	1,440,608	(49%)	2,895,781	1,568,840	(54%)	2,870,920	1,462,230	(51%)
Household member	1,539,092	801,182	(52%)	1,451,286	858,167	(59%)	1,456,171	921,859	(63%)	1,446,208	849,220	(59%)
Enterprise size (no. of employees)												
≥ 300	4,467,423	3,546,046	(79%)	4,258,740	3,482,842	(82%)	4,628,576	3,786,158	(82%)	4,549,044	3,748,268	(82%)
50–299	2,893,594	2,371,029	(82%)	2,694,642	2,268,129	(84%)	2,927,294	2,455,925	(84%)	2,845,016	2,400,555	(84%)
< 50	4,874,679	3,422,505	(70%)	5,017,236	3,689,450	(74%)	4,823,102	3,350,385	(69%)	5,176,526	3,625,079	(70%)
Sex												
Male	9,201,725	6,323,006	(69%)	8,814,474	6,443,675	(73%)	8,985,416	6,603,480	(73%)	9,049,455	6,613,771	(73%)
Female	7,838,049	5,168,724	(66%)	7,518,990	5,296,099	(70%)	7,745,624	5,479,796	(71%)	7,862,009	5,495,114	(70%)
Age												
20s	1,495,971	1,232,879	82%)	1,464,287	1,245,499	(85%)	1,361,209	1,147,620	(84%)	1,260,888	1,062,788	(84%)
30s	3,075,001	2,240,262	(73%)	2,897,369	2,213,257	(76%)	2,933,731	2,250,399	(77%)	2,764,869	2,143,148	(78%)
40s	4,791,994	3,017,490	(63%)	4,516,171	3,071,894	(68%)	4,525,205	3,110,001	(69%)	4,684,605	3,206,437	(68%)
50s	3,859,138	2,598,385	(67%)	3,757,946	2,710,203	(72%)	3,986,889	2,895,631	(73%)	4,080,043	2,896,439	(71%)
60s	2,140,255	1,513,228	(71%)	2,000,631	1,517,664	(76%)	2,123,343	1,610,092	(76%)	2,224,283	1,681,885	(76%)
70s	1,245,070	746,457	(60%)	1,263,309	815,426	(65%)	1,344,136	885,701	(66%)	1,394,240	917,989	(66%)
80s	366,394	118,893	(32%)	364,581	136,226	(37%)	380,969	149,655	(39%)	427,317	172,632	(40%)
90s	44,850	6,122	(14%)	45,030	7,667	(17%)	47,351	8,336	(18%)	56,214	11,183	(20%)

^a^TS: Total subjects

^b^P: Participants in health examination

To indicate the current status of the WGHE, we examined changes in the participation rates of the NGHE over the study period. Furthermore, we described the participation rates of the primary survey with those of the secondary survey, and determined the differences in general participation rates by sex, age, insurance type, and enterprise size. Regarding insurance type, the levels considered included workplace policyholders among office workers or non-office workers, dependents of workplace policyholders aged 40 or older, regional policyholders who are householders, and household members aged 40 or older. Regarding enterprise size—which was determined only among workplace policyholders—workplaces were divided according to the number of full-time employees: 300 or more, between 50 and 299, and less than 50.

After determining the proportion of examinees with abnormal results indicating either hypertension or diabetes at each workplace (abnormal results for high blood pressure and high blood glucose were determined by the physicians at the facilities where examinees had received the health examination) and determining differences in these proportions by age, insurance type, and enterprise size, we calculated the odds ratios of having hypertension or diabetes by age and enterprise size using logistic regression analysis. For the odds ratios according to enterprise size, workplaces with 300 or more employees were used as the reference. This analysis was performed to identify possible health disparities due to socioeconomic status. All statistical analyses were conducted using SAS Enterprise 4.3 (SAS Institute, Cary, NC, USA).

## Results

### Distribution of primary examination participation rates

Table 1 shows the distribution of participation rates in the WGHE conducted by the National Health Insurance Service between 2006 and 2013. The total number of examinees was 15,053,761 in 2006 and 16,911,464 in 2013. Thus, an estimated 14–17 million individuals took part. In contrast, the number of participants in the NGHE was 8,408,218 in 2006 and 12,108,885 in 2013. The overall participation rates were as low as 43% in 2002 (not presented in the table), but increased thereafter, reaching 56% in 2006 and 72% in 2011 (the first point at which it exceeded 70%), at which point it leveled off (remaining 72% in 2012 and 2013).

We also observed differences in participation rates depending on insurance type, sex, and age. Regarding sex differences, the participation rate of male examinees (58%) was higher than that of female examinees (53%) in 2006. However, as the overall participation rate increased, the gap between the sexes decreased, reaching 73% and 70% among male and female examinees, respectively, in 2013.

As for age groups, the highest participation rates were observed among examinees in their 20s (77%) in 2006, followed by 30s (63%), 50s (56%), 60s (55%), 40s (49%), and 70s (44%) in that order. In 2013, the participation rates were ranked (from highest to lowest) as 20s (84%), 30s (78%), 60s (76%), 50s (71%), 40s (68%), and 70s (66%).

Regarding insurance type, workplace policyholders had the highest participation rates. In 2006, the participation rate for “non-public workers” overall was 77%. When these examinees were divided to office and non-office workers, the participation rate of non-office workers was higher, at 80%. In 2013, the participation rate of workplace policyholders was 83%, with that of non-office workers being 91%. All of these figures were higher than were those of regional policyholders, whose participation rate was less than 60% by 2013, despite the fact the general participation rates have continued increasing since 2006. In 2013, the participation rate of regional policyholders who were householders was 51%, while that of household members aged 40 or older was 59%. The rate among workplace policyholders’ dependents aged 40 or older was 66% in 2013; as can be seen, these figures are higher than are those of regional policyholders but lower than are those of workplace policyholders.

Regarding enterprise size among workplace policyholders, workers in enterprises with 50 to 299 employees showed the highest participation rates (77% and 84% in 2006 and 2013, respectively), and the rates gradually increased between 2006 and 2013. The second highest participation rates were shown among workplaces with 300 or more employees (74% and 82% in 2006 and 2013, respectively), and these rates gradually increased as well. Workplace policyholders at enterprises with less than 50 employees showed the lowest participation rates, at 67% and 70% in 2006 and 2013, respectively. The rates differed significantly from those of workers at enterprises with 50 or more employees.

### Distribution of secondary examination participation rates

Table 2 shows the distribution of second examination participation rates. Data were available for about 0.99 million to 1.7 million individuals (see Table 2). In general, the participation rates were lower than were those of the primary health examination. Furthermore, despite the growth of primary examination participation rates over time, we observed no significance change or noticeable trend in secondary examination participation rates over the study period: in 2006, the rate was 35%, which then decreased to 30% in 2009 and increased again to 36% in 2013.

**Table 2:** Participation rates in secondary health examination

	2006			2007			2008				2009		
	TS^a^ (N)	P^b^ (n, %)		TS (N)	P (n, %)		TS (N)		P (n, %)		TS (N)	P (n, %)	
Overall participation rate	1,528,258	542,159	(35%)	1,510,570	494,307	(33%)	1,685,419		613,629	(36%)	1,338,668	531,368	(40%)
National health insurance type													
Workplace policyholder													
Non-public worker	653,174	322,341	(49%)	665,972	302,288	(45%)	762,107		349,892	(46%)	770,863	353,721	(46%)
Public official	101,161	30,023	(30%)	32,539	8,961	(28%)	88,985		28,977	(33%)	24,140	7,742	(32%)
Dependents	391,370	97,262	(25%)	442,657	97,755	(22%)	454,519		124,020	(27%)	183,432	58,923	(32%)
Regional policyholder													
Householder	243,231	59,889	(25%)	238,327	56,214	(24%)	246,343		73,685	(30%)	236,901	74,466	(31%)
Household member	139,322	32,644	(23%)	131,075	29,089	(22%)	133,465		37,055	(28%)	123,332	36,516	(30%)
Enterprise size (no. of employees)													
≥ 300	275,350	136,199	(49%)	225,949	112,065	(50%)	268,608		129,066	(48%)	219,811	113,998	(52%)
50–299	219,415	115,623	(53%)	197,555	101,858	(52%)	235,050		121,476	(52%)	209,477	115,495	(55%)
< 50	269,550	104,625	(39%)	289,385	102,813	(36%)	358,311		133,238	(37%)	377,482	137,729	(36%)
Sex													
Male	978,849	389,925	(40%)	946,445	352,068	(37%)	1,073,130		427,195	(40%)	1,018,462	419,269	(41%)
Female	549,409	152,234	(28%)	564,125	142,239	(25%)	612,289		186,434	(30%)	320,206	112,099	(35%)
Age													
20s	53,692	29,402	(55%)	53,881	28,447	(53%)	59,137		31,297	(53%)	51,077	25,389	(50%)
30s	182,018	87,805	(48%)	174,085	82,411	(47%)	200,580		94,595	(47%)	173,040	79,718	(46%)
40s	349,126	140,327	(40%)	320,442	115,845	(36%)	399,238		148,974	(37%)	326,432	135,562	(42%)
50s	399,951	144,490	(36%)	381,998	137,387	(36%)	488,436		189,839	(39%)	392,327	154,592	(39%)
60s	343,325	97,380	(28%)	353,618	84,869	(24%)	355,684		102,449	(29%)	349,726	125,906	(36%)
70s	173,630	38,075	(22%)	192,742	40,215	(21%)	157,251		41,561	(26%)	233,235	71,208	(31%)
80s	25,238	4,363	(17%)	32,230	4,844	(15%)	23,541		4,619	(20%)	92,784	22,048	(24%)
90s	1,026	149	(15%)	1,327	138	(10%)	1,295		172	(13%)	13,937	2,607	(19%)
	2010			2011			2012				2013		
	TS (N)	P (n, %)		TS (N)	P (n, %)		TS (N)		P (n, %)		TS (N)	P (n, %)	
Overall participation rate	1,013,976	414,029	(41%)	997,658	372,073	(37%)	1,154,033	429,375	(37%)		1,078,029	390,170	(36%)
National health insurance type													
Workplace policyholder													
Non-public worker	597,762	280,244	(47%)	607,131	265,565	(44%)	576,320	267,880	(46%)		571,016	249,827	(44%)
Public official	46,354	14,505	(31%)	22,112	5,313	(24%)	44,789	12,177	(27%)		21,650	5,225	(24%)
Dependents	114,741	38,049	(33%)	110,150	31,263	(28%)	256,002	73,162	(29%)		247,981	69,859	(28%)
Regional policyholder													
Householder	175,332	56,573	(32%)	177,090	48,673	(27%)	190,654	52,647	(28%)		164,155	45,594	(28%)
Household member	79,787	24,658	(31%)	81,175	21,259	(26%)	86,268	23,509	(27%)		73,227	19,665	(27%)
Enterprise size (no. of employees)													
≥ 300	206,469	103,135	(50%)	189,163	94,346	(50%)	210,284	104,726	(50%)		189,634	96,030	(51%)
50–299	174,351	94,622	(54%)	163,767	87,370	(53%)	173,687	92,500	(53%)		159,209	81,765	(51%)
< 50	270,055	99,992	(37%)	281,230	91,208	(32%)	239,642	84,064	(35%)		245,452	78,007	(32%)
Sex													
Male	797,375	336,353	(42%)	784,090	304,117	(39%)	789,236	317,283	(40%)		733,806	286,863	(39%)
Female	216,601	77,676	(36%)	213,568	67,956	(32%)	364,797	112,092	(31%)		344,223	103,307	(30%)
Age													
20s	42,001	21,505	(51%)	41,116	19,830	(48%)	36,470	17,710	(49%)		30,385	13,873	(46%)
30s	160,820	74,726	(46%)	162,101	70,814	(44%)	155,709	71,811	(46%)		137,908	61,367	(44%)
40s	303,173	122,650	(40%)	306,178	112,285	(37%)	303,601	116,296	(38%)		288,295	104,637	(36%)
50s	332,857	133,182	(40%)	331,139	120,083	(36%)	341,649	129,235	(38%)		316,123	117,016	(37%)
60s	208,220	77,363	(37%)	190,034	61,912	(33%)	190,899	65,006	(34%)		184,237	63,703	(35%)
70s	102,762	28,613	(28%)	99,100	24,670	(25%)	104,077	26,562	(26%)		98,095	26,123	(27%)
80s	17,925	3,388	(19%)	18,035	2,988	(17%)	19,829	2,424	(12%)		20,917	3,141	(15%)
90s	1,093	141	(13%)	1,261	157	(12%)	1,362	90	(7%)		1,762	183	(10%)

^a^TS: Total subjects

^b^P: Participants in health examination

Regarding the differences by sex, the participation rates of male and female examinees was 40% and 28% in 2006, respectively; the highest participation rates were 42% and 36% in 2010, although they decreased to 39% and 30%, respectively, in 2013. As for age groups, the participation rates of examinees in their 20s were consistently the highest between 2006 and 2013, with the rates decreasing as age increased.

Regarding the insurance types, the participation rates of workplace policyholders were consistently the highest, as with the primary examination. However, the participation rates were lower than 50% in 2006 and 2013, at 49 and 44%, respectively. The participation rates of public officials, regional policyholders, and dependents were as low as 22–33%, and no significant difference was observed during the observation period.

For the enterprise size comparison, the secondary examination participation rates showed no significant differences by enterprise size and a non-significant increase by year. However, the workers at enterprises with less than 50 employees, which were generally lower than the participation rates of those with 50 or more employees, showed decreases in participation over time. Specifically, the participation rates at enterprises with less than 50 employees were 39% in 2006 and 32% in 2013. During the same periods, participation rates at enterprises with 50 to 299 employees were 53 and 51%, while those at enterprises with 300 or more employees were 49 and 51%, respectively. Thus, the gap in secondary examination participation rates between enterprises with less than 50 and those with 50 or more employees was considerable, as with the primary examination participation rates.

### Prevalence rates of abnormal results among health screening examinees of the national health insurance service

Table 3 shows the distribution of examinees with abnormal results indicating diabetes and hypertension. Overall, the prevalence rates of diabetes and hypertension of the total were around 3 and 7% in 2006, respectively, and 6 and 17% in 2013.

**Table 3:** Prevalence rates of diabetes and hypertension among study population

	2005					2006															
		DM		HTN			Diabetes			Hypertension											
	Total subjects(N)	Cases (N,%)		Cases (N,%)		Total (N)	Cases (n, %)			Cases (n, %)											
Total prevalence																					
	6,399,291	190,937	(3%)	462,760	(7%)	8,408,218	261,216	3.11%	(3%)	668,277	7.9%	(8%)									
Nation health insurance type																					
Regional policyholder + dependents	2,305,435	134,744	(6%)	349,530	(15%)	2,880,225	179,950	6.25%	(6%)	484,164	16.8%	(17%)									
Non-public worker																					
Office worker	687,522	8,630	(1%)	15,481	(2%)	1,078,249	15,453	1.43%	(1%)	32,584	3.0%	(3%)									
Non-office worker	3,251,609	44,261	(1%)	91,635	(3%)	3,677,914	51,862	1.41%	(1%)	118,429	3.2%	(3%)									
Public official	154,725	3,302	(2%)	6,114	(4%)	771,830	13,951	1.81%	(2%)	33,100	4.3%	(4%)									
Enterprise size																					
≥ 300	2,044,945	48,381	(2%)	114,524	(6%)	2,825,502	69,946	2.48%	(2%)	176,557	6.25%	(6%)									
50–299	1,387,051	33,339	(2%)	78,854	(6%)	1,905,700	46,342	2.43%	(2%)	117,696	6.18%	(6%)									
< 50	1,749,381	45,211	(3%)	110,543	(6%)	2,179,986	59,908	2.75%	(3%)	154,446	7.08%	(7%)									
Missing	1,217,914	64,006		158,839		1,497,030	85,020	5.68%		219,578	14.67%										
Sex																					
Male	3,767,406	108,226	(3%)	216,368	(6%)	4,858,065	147,842	3.04%	(3%)	318,606	6.56%	(7%)									
Female	2,631,885	82,711	(3%)	246,392	(9%)	3,550,153	113,374	3.19%	(3%)	349,671	9.85%	(10%)									
Age																					
20s	927,532	1,864	(0%)	1,671	(0%)	1,188,441	2,179	0.18%	(0%)	2,185	0.18%	(0%)									
30s	1,450,646	8,309	(1%)	11,403	(1%)	1,874,052	9,663	0.52%	(1%)	15,952	0.85%	(1%)									
40s	1,682,743	30,054	(2%)	60,911	(4%)	2,252,394	39,624	1.76%	(2%)	90,148	4.00%	(4%)									
50s	1,187,314	52,803	(4%)	128,562	(11%)	1,608,651	73,793	4.59%	(5%)	189,496	11.78%	(12%)									
60s	778,422	64,175	(8%)	159,391	(20%)	984,003	86,939	8.84%	(9%)	222,100	22.57%	(23%)									
70s	313,968	30,304	(10%)	87,743	(28%)	426,452	44,233	10.37%	(10%)	129,941	30.47%	(30%)									
80s	44,933	3,344	(7%)	12,732	(28%)	56,875	4,682	8.23%	(8%)	17,922	31.51%	(32%)									
90s	1,587	63	(4%)	339	(21%)	2,142	76	3.55%	(4%)	525	24.51%	(25%)									
Missing	12,146	12,146		12,146		15,208	27			8											
	2007							2008													
		Diabetes			Hypertension				Diabetes			Hypertension									
	Total (N)	Cases (n,%)			Cases (n,%)			Total (N)	Cases (n,%)			Cases (n,%)									
Total prevalence																					
	8,419,532	265,813	3.16%	(3%)	708,647	8.42%	(8%)	10,264,420	331,893	3.23%	(3%)	900,056	8.77%	(9%)							
Nation health insurance type																					
Regional policyholder + dependents	3,189,796	190,808	5.98%	(6%)	528,428	16.57%	(17%)	3,531,109	221,581	6.28%	(6%)	619,038	17.53%	(18%)							
Non-public worker																					
Office worker	839,517	10,055	1.20%	(1%)	21,177	2.52%	(3%)	2,044,152	32,126	1.57%	(2%)	77,571	3.79%	(4%)							
Non-office worker	4,176,693	60,694	1.45%	(1%)	149,595	3.58%	(4%)	3,909,321	63,217	1.62%	(2%)	164,400	4.21%	(4%)							
Public official	213,526	4,256	1.99%	(2%)	9,447	4.42%	(4%)	779,838	14,969	1.92%	(2%)	39,047	5.01%	(5%)							
Enterprise size																					
≥ 300	2,663,471	68,577	2.57%	(3%)	183,148	6.88%	(7%)	3,195,498	86,921	2.72%	(3%)	235,100	7.36%	(7%)							
50–299	1,768,735	46,586	2.63%	(3%)	121,795	6.89%	(7%)	2,235,709	59,410	2.66%	(3%)	161,778	7.24%	(7%)							
< 50	2,443,371	67,316	2.76%	(3%)	182,838	7.48%	(7%)	3,133,715	90,022	2.87%	(3%)	247,560	7.90%	(8%)							
missing	1,543,955	83,334	5.40%		220,866	14.31%		1,699,498	95,540	5.62%		255,618	15.04%								
Sex																					
Male	4,833,287	146,782	3.04%	(3%)	328,619	6.80%	(7%)	5,763,063	188,671	3.27%	(3%)	435,431	7.56%	(8%)							
Female	3,586,245	119,031	3.32%	(3%)	380,028	10.60%	(11%)	4,501,357	143,222	3.18%	(3%)	464,625	10.32%	(10%)							
Age																					
20s	1,199,391	1,919	0.16%	(0%)	2,264	0.19%	(0%)	1,413,162	2,086	0.15%	(0%)	2,905	0.21%	(0%)							
30s	1,764,649	9,132	0.52%	(1%)	16,115	0.91%	(1%)	2,170,990	10,824	0.50%	(0%)	21,503	0.99%	(1%)							
40s	2,156,367	35,167	1.63%	(2%)	84,647	3.93%	(4%)	2,677,379	45,085	1.68%	(2%)	113,828	4.25%	(4%)							
50s	1,620,394	75,899	4.68%	(5%)	200,596	12.38%	(12%)	2,067,020	101,633	4.92%	(5%)	273,742	13.24%	(13%)							
60s	1,081,039	81,470	7.54%	(8%)	214,586	19.85%	(20%)	1,260,900	93,829	7.44%	(7%)	251,245	19.93%	(20%)							
70s	504,698	55,267	10.95%	(11%)	163,285	32.35%	(32%)	575,863	70,028	12.16%	(12%)	204,293	35.48%	(35%)							
80s	77,305	6,805	8.80%	(9%)	26,294	34.01%	(34%)	82,816	8,214	9.92%	(10%)	31,491	38.03%	(38%)							
90s	3,063	140	4.57%	(5%)	850	27.75%	(28%)	3,276	175	5.34%	(5%)	1,043	31.84%	(32%)							
Missing	12,626	14			10			13,014	19			6									
	2009							2010													
	Diabetes				Hypertension			Diabetes				Hypertension									
	Total (n)	Cases (n, %)			Cases (n, %)			Total (n)	Cases (n, %)			Cases (n, %)									
Total prevalence																					
	10,537,866	535,904	5.09%	(5%)	1,481,623	14.06%	(14%)	11,491,730	612,976	5.33%	(5%)	1,762,190	15.33%	(15%)							
Nation health insurance type																					
Regional policyholder + dependents	4,051,519	345,125	8.52%	(9%)	971,687	23.98%	(24%)	4,470,443	411,405	9.20%	(9%)	1,164,126	26.04%	(26%)							
Non-public worker																					
Office worker	2,049,755	56,098	2.74%	(3%)	155,502	7.59%	(8%)	2,088,031	63,998	3.06%	(3%)	177,668	8.51%	(9%)							
Non-office worker	4,240,682	129,358	3.05%	(3%)	340,193	8.02%	(8%)	4,326,907	122,166	2.82%	(3%)	373,474	8.63%	(9%)							
Public official	195,910	5,323	2.72%	(3%)	14,241	7.27%	(7%)	606,349	15,407	2.54%	(3%)	46,922	7.74%	(8%)							
Enterprise size																					
≥ 300	3,119,928	153,087	4.91%	(5%)	397,562	12.74%	(13%)	3,546,046	162,448	4.58%	(5%)	466,600	13.16%	(13%)							
50–299	2,125,705	88,321	4.15%	(4%)	259,239	12.20%	(12%)	2,371,029	104,584	4.41%	(4%)	314,656	13.27%	(13%)							
< 50	3,456,632	157,402	4.55%	(5%)	454,658	13.15%	(13%)	3,422,505	172,856	5.05%	(5%)	509,232	14.88%	(15%)							
Missing	1,835,601	137,094	7.47%		370,164	20.17%		2,152,150	173,088	8.04%		471,702	21.92%								
Sex																					
Male	5,816,276	308,519	5.30%	(5%)	742,507	12.77%	(13%)	6,323,006	352,891	5.58%	(6%)	907,367	14.35%	(14%)							
Female	4,721,590	227,385	4.82%	(5%)	739,116	15.65%	(16%)	5,168,724	260,085	5.03%	(5%)	854,823	16.54%	(17%)							
Age																					
20s	1,305,813	3,015	0.23%	(0%)	10,025	0.77%	(1%)	1,232,879	2,055	0.17%	(0%)	8,477	0.69%	(1%)							
30s	2,014,012	18,180	0.90%	(1%)	46,242	2.30%	(2%)	2,240,262	16,926	0.76%	(1%)	48,791	2.18%	(2%)							
40s	2,744,518	82,776	3.02%	(3%)	198,893	7.25%	(7%)	3,017,490	81,254	2.69%	(3%)	220,462	7.31%	(7%)							
50s	2,235,227	146,546	6.56%	(7%)	407,900	18.25%	(18%)	2,598,385	176,686	6.80%	(7%)	513,783	19.77%	(20%)							
60s	1,421,468	170,971	12.03%	(12%)	471,277	33.15%	(33%)	1,513,228	197,633	13.06%	(13%)	553,203	36.56%	(37%)							
70s	692,595	101,393	14.64%	(15%)	297,958	43.02%	(43%)	746,457	121,671	16.30%	(16%)	354,564	47.50%	(47%)							
80s	104,206	12,659	12.15%	(12%)	47,414	45.50%	(46%)	118,893	16,238	13.66%	(14%)	60,176	50.61%	(51%)							
90s	4,676	338	7.23%	(7%)	1,843	39.41%	(39%)	6,122	479	7.82%	(8%)	2,648	43.25%	(43%)							
Missing	15,351	26			71			18,014	34			86									
	2011							2012							2013						
	Diabetes				Hypertension			Diabetes				Hypertension			Diabetes				Hypertension		
	Total (N)	Cases (n, %)			Cases (n, %)			Total (N)	Cases (n, %)			Cases (n, %)			Total (N)	Cases (n, %)			Cases (n, %)		
Total prevalence																					
	11,739,774	660,173	5.62%	(6%)	1,894,835	16.14%	(16%)	12,083,276	720,785	5.97%	(6%)	2,036,249	16.85%	(17%)	12,108,885	752,479	6.21%	(6%)	2,092,682	17.28%	(17%)
Nation health insurance type																					
Regional policyholder + dependents	4,743,687	453,771	9.57%	(10%)	1,268,580	26.74%	(27%)	4,935,574	500,276	10.14%	(10%)	1,366,098	27.68%	(28%)	4,875,356	516,196	10.59%	(11%)	1,392,427	28.56%	(29%)
Non-public worker																					
Office worker	2,302,668	67,656	2.94%	(3%)	191,364	8.31%	(8%)	2,120,013	67,636	3.19%	(3%)	192,517	9.08%	(9%)	2,091,641	66,376	3.17%	(3%)	182,837	8.74%	(9%)
Non-office worker	4,442,913	130,895	2.95%	(3%)	413,879	9.32%	(9%)	4,458,500	135,218	3.03%	(3%)	425,481	9.54%	(10%)	4,860,568	160,762	3.31%	(3%)	492,731	10.14%	(10%)
Public official	250,506	7,851	3.13%	(3%)	21,012	8.39%	(8%)	569,189	17,655	3.10%	(3%)	52,153	9.16%	(9%)	281,320	9,145	3.25%	(3%)	24,687	8.78%	(9%)
Enterprise size																					
≥ 300	3,482,842	163,177	4.69%	(5%)	486,099	13.96%	(14%)	3,786,158	185,987	4.91%	(5%)	543,384	14.35%	(14%)	3,748,268	190,627	5.09%	(5%)	548,147	14.62%	(15%)
50–299	2,268,129	110,318	4.86%	(5%)	326,985	14.42%	(14%)	2,455,925	123,225	5.02%	(5%)	360,713	14.69%	(15%)	2,400,555	128,615	5.36%	(5%)	367,928	15.33%	(15%)
< 50	3,689,450	193,974	5.26%	(5%)	564,875	15.31%	(15%)	3,350,385	189,917	5.67%	(6%)	553,200	16.51%	(17%)	3,625,079	215,483	5.94%	(6%)	610,829	16.85%	(17%)
Missing	2,299,353	192,704	8.38%		516,876	22.48%		2,490,808	221,656	8.90%		578,952	23.24%		2,334,983	217,754	9.33%		565,778	24.23%	
Sex																					
Male	6,443,675	375,531	5.83%	(6%)	972,053	15.09%	(15%)	6,603,480	414,207	6.27%	(6%)	1,064,970	16.13%	(16%)	6,613,771	430,830	6.51%	(7%)	1,092,632	16.52%	(17%)
Female	5,296,099	284,642	5.37%	(5%)	922,782	17.42%	(17%)	5,479,796	306,578	5.59%	(6%)	971,279	17.72%	(18%)	5,495,114	321,649	5.85%	(6%)	1,000,050	18.20%	(18%)
Age																					
20s	1,245,499	2,072	0.17%	(0%)	8,713	0.70%	(1%)	1,147,620	1,908	0.17%	(0%)	7,648	0.67%	(1%)	1,062,788	1,837	0.17%	(0%)	6,964	0.66%	(1%)
30s	2,213,257	15,526	0.70%	(1%)	51,580	2.33%	(2%)	2,250,399	15,710	0.70%	(1%)	50,231	2.23%	(2%)	2,143,148	13,730	0.64%	(1%)	45,107	2.10%	(2%)
40s	3,071,894	82,047	2.67%	(3%)	229,048	7.46%	(7%)	3,110,001	85,978	2.76%	(3%)	238,180	7.66%	(8%)	3,206,437	87,144	2.72%	(3%)	235,812	7.35%	(7%)
50s	2,710,203	190,651	7.03%	(7%)	550,829	20.32%	(20%)	2,895,631	206,781	7.14%	(7%)	591,902	20.44%	(20%)	2,896,439	208,241	7.19%	(7%)	587,063	20.27%	(20%)
60s	1,517,664	207,082	13.64%	(14%)	572,466	37.72%	(38%)	1,610,092	223,860	13.90%	(14%)	610,749	37.93%	(38%)	1,681,885	238,413	14.18%	(14%)	638,455	37.96%	(38%)
70s	815,426	141,384	17.34%	(17%)	405,298	49.70%	(50%)	885,701	161,244	18.21%	(18%)	449,970	50.80%	(51%)	917,989	172,334	18.77%	(19%)	475,139	51.76%	(52%)
80s	136,226	20,679	15.18%	(15%)	73,267	53.78%	(54%)	149,655	24,369	16.28%	(16%)	83,411	55.74%	(56%)	172,632	29,530	17.11%	(17%)	98,320	56.95%	(57%)
90s	7,667	687	8.96%	(9%)	3,523	45.95%	(46%)	8,336	874	10.48%	(10%)	4,047	48.55%	(49%)	11,183	1,220	10.91%	(11%)	5,749	51.41%	(51%)
Missing	21,938	45			111			25,841	61			111			16,384	30			73		

Regarding the differences by sex, we observed no significant differences in the prevalence rate of diabetes, although male examinees had a slightly higher rate. In contrast, the rate of hypertension was higher among female examinees than among male examinees. Notably, the rates increased in proportion with age.

As for insurance types, the ratio of regional policyholders was higher than that of workplace policyholders. However, it must be taken into account that the ratios of working ages (i.e., those under the retirement age) were relatively higher among workplace policyholders, while the ratio of elderly persons aged 60 or older was higher among regional policyholders (not presented in the table), which could have affected the prevalence rate results (given that older adults are more likely to have blood pressure and blood glucose abnormalities). Table 3 also shows the ratios of examinees with abnormal results among workplace policyholders who were office workers, non-office workers, and public officials, as well as the ratios depending on enterprise size. Except for the ratios of diabetes in 2009, workers at enterprises with less than 50 employees showed higher ratios of abnormal results of diabetes and hypertension than did workers at workplaces with 50 or more employees.

### Odds ratios of abnormal results among workplace policyholders depending on enterprise size

Table 4 shows the odds ratios of abnormal results of diabetes and hypertension among workers in their 40s and 50s by enterprise size, with enterprises of 300 or more employees as the reference. The overall odds ratios of diabetes among those in their 40s and 50s, along with odds ratios of diabetes among those in their 40s in 2009 and 2010, were all less than 1. Among the remaining examinees, the odds ratios of diabetes and hypertension among workers at enterprises with less than 300 employees tended to be higher than did those among workers at enterprises with 300 or more employees. More specifically, workers at enterprises with less than 50 employees showed the highest odds ratios for both diabetes and hypertension compared to workers at enterprises with 50 or more employees.

**Table 4:** Odds ratios of diabetes and hypertension among workplace policyholders in their 40s and 50s by enterprise size (2006–2013)

	Diabetes				Hypertension			
	40s		50s		40s		50s	
Year Enterprise size (no. of employees)	OR	95% CI	OR	95% CI	OR	95% CI	OR	95% CI
2006								
≥ 300	1.0 (ref)		1.0 (ref)		1.0 (ref)		1.0 (ref)	
50 ~ 299	1.071	(1.04 – 1.102)	1.022	(0.999 – 1.045)	1.093	(1.071 – 1.114)	1.041	(1.026 – 1.057)
< 50	1.204	(1.171 – 1.238)	1.07	(1.046 – 1.093)	1.259	(1.236 – 1.283)	1.108	(1.092 – 1.124)
2007								
≥ 300	1.0 (ref)		1.0 (ref)		1.0 (ref)		1.0 (ref)	
50 ~ 299	1.169	(1.132 – 1.207)	1.107	(1.082 – 1.133)	1.163	(1.138 – 1.188)	1.062	(1.046 – 1.078)
< 50	1.253	(1.216 – 1.29)	1.129	(1.105 – 1.153)	1.346	(1.321 – 1.373)	1.155	(1.14 – 1.171)
2008								
≥ 300	1.0 (ref)		1.0 (ref)		1.0 (ref)		1.0 (ref)	
50 ~ 299	1.041	(1.012 – 1.071)	1.036	(1.016 – 1.057)	1.107	(1.088 – 1.127)	1.036	(1.024 – 1.049)
< 50	1.168	(1.138 – 1.199)	1.074	(1.055 – 1.094)	1.264	(1.243 – 1.285)	1.116	(1.103 – 1.129)
2009								
≥ 300	1.0 (ref)		1.0 (ref)		1.0 (ref)		1.0 (ref)	
50 ~ 299	0.555	(0.543 – 0.567)	0.955	(0.939 – 0.971)	0.837	(0.826 – 0.849)	1.011	(1.001 – 1.022)
< 50	0.615	(0.604 – 0.627)	0.976	(0.962 – 0.991)	0.898	(0.887 – 0.909)	1.052	(1.042 – 1.062)
2010								
≥ 300	1.0 (ref)		1.0 (ref)		1.0 (ref)		1.0 (ref)	
50 ~ 299	0.907	(0.888 – 0.926)	1.028	(1.013 – 1.044)	1.071	(1.057 – 1.085)	1.037	(1.027 – 1.047)
< 50	0.97	(0.952 – 0.988)	1.058	(1.044 – 1.072)	1.131	(1.118 – 1.144)	1.068	(1.059 – 1.077)
2011								
≥ 300	1.0 (ref)		1.0 (ref)		1.0 (ref)		1.0 (ref)	
50 ~ 299	1.124	(1.121 – 1.128)	1.097	(1.095 – 1.099)	1.085	(1.071 – 1.099)	1.065	(1.055 – 1.075)
< 50	2.535	(2.483 – 2.588)	1.996	(1.971 – 2.022)	1.114	(1.102 – 1.127)	1.078	(1.069 – 1.088)
2012								
≥ 300	1.0 (ref)		1.0 (ref)		1.0 (ref)		1.0 (ref)	
50 ~ 299	1.052	(1.031 – 1.074)	1.063	(1.048 – 1.078)	1.071	(1.058 – 1.084)	1.062	(1.052 – 1.071)
< 50	1.098	(1.078 – 1.119)	1.048	(1.035 – 1.061)	1.113	(1.101 – 1.126)	1.069	(1.06 – 1.077)
2013								
≥ 300	1.0 (ref)		1.0 (ref)		1.0 (ref)		1.0 (ref)	
50 ~ 299	1.047	(1.026 – 1.069)	1.109	(1.093 – 1.125)	1.063	(1.05 – 1.076)	1.087	(1.077 – 1.096)
< 50	1.082	(1.063 – 1.102)	1.082	(1.068 – 1.096)	1.087	(1.075 – 1.1)	1.092	(1.083 – 1.101)

## Discussion

In the following section, we explore the history of the Korean health screening system and the present national health screening and workers’ health examinations. We believe that, to understand the modern Korean health screening system, it is necessary to look back on the history of health screening. This will provide some information on how the current policies, institutions, and attitudes and culture of the people concerning this topic have been formed.

### History of health examination and domestic system of national general health screening

Although the specific origin of organized health examinations (OHEs) is uncertain, the first recorded regular health examination/screening dates back to the 14th century [8]. In modern medical science, the concept of performing a health screening for those without particular symptoms was first suggested by Horace Dobell, a medical scientist in the UK [9]. In the US, the first health screenings were conducted in the mid-19th century among immigrants in line with quarantine inspections [10]. In the 20th century, Gould suggested the necessity of OHEs for general population groups, becoming the first promoter of general health examinations in the formal academic literature [11, 12]. Until the early 20th century, health examinations were conducted in the western world mainly in order to control outbreaks of infectious diseases such as tuberculosis [13].

As the number of patients with tuberculosis decreased in the late 1950s, more doctors began proposing that the scope of disease prevention through health examination should be broadened to cover chronic diseases [14, 15]. Thereafter, changes in the roles of medical doctor groups [16] and life insurance companies, the emergence of preventive medicine, the lingering effects of the World Wars, etc., [17] resulted in an expansion of health examinations throughout various areas of society. In the 1960s, several critical and scientific studies revealed that many elements of the health examinations conducted at those times were not scientifically verified in terms of their predictive ability of treatment outcomes [18–20]. This led to a number of the more detailed questions and physical examinations, which had once been key elements of regular health examinations, no longer being recommended. Thereafter, general medical preventive examinations conducted as part of the national insurance system for the general population became relatively rare in western countries [10].

Given this background, it can be said that the Korean NGHE is a rather unique medical service around the world. The concept of health examination for groups without specific symptoms was first introduced in the Republic of Korea around the time of the national liberation in 1945. After the liberation in 1945, health examinations began being institutionalized, starting with the examination of parasites and infectious diseases such as tuberculosis [21]. The national health examination system was initiated as part of the national medical insurance in 1977; before this, health examinations were conducted individually according to specific needs and subjects.

From 1977, all companies with more than 500 employees were required to provide health insurance services. The health insurance coverage expanded to cover companies with 300 or more employees, public workers, and school personnel in 1979, and thereafter its coverage expanded to even smaller companies. Finally, the self-employed were included under national health insurance coverage in 1989 as regional policyholders. Since 1995, national medical insurance covered both public health services and national health screening. However, between 1977 and 1995, the Ministry of Labor took charge of both the WGHE and WSHE; during this period, the national health screening service could not be used as a substitute for the WGHE. In 1995, authority of the health examination service was transferred to the Ministry of Health and Welfare as part of the National Health Promotion Act; thereafter, health insurance policyholders were able to claim health examination services under their insurance coverage [21]. The employers’ obligation of reporting the results of the WGHE to local branch of the Ministry of Labor ceased in 1997, while the obligation of reporting to the Korea Occupational Safety and Health Agency (KOSHA) was ceased in 2005 [22]. All health insurance societies were integrated into a single insurer, the National Health Insurance Program, in 2000 [23]. The health examination system has been continually expanded with successive acts since, including the Framework Act on Health Examination in 2008, the 1^st^ 5-year plan for the NGHE in 2010, etc., and with the establishment of the 2^nd^ 5-year plan for the NGHE in 2016. This act has led to the current examination system and framework of operation [1, 24].

### Beginning of current WGHE and participation rates of workplace policyholders

The NGHE, which is provided to workplace policyholders (i.e., beneficiaries of national health insurance), is a replacement of the WGHE originally specified as a duty of business owners for employee health protection designated by the Industrial Safety and Health Act. One major historical change in the domestic health examination system for workers [25] was the Labor Standard Act’s stipulation in 1953 that enterprises with 16 or more employees must provide regular health examinations for workers. The major subjects of this legislation were miners. In 1961, regulations on workers’ health management were announced, specifying the types, subjects, contents, and intervals of the health examinations. Then, in 1972, health examinations were divided into the WSHE and WGHE, depending on the hazardous substances that workers would be exposed to in their workplaces. The enforcement ordinances of the Industrial Safety and Health Act were subsequently revised in 1981, which stipulated that business owners of business entities with 5 or more employees were obligated to provide workers with health examination services. This also led office workers to being distinguished from non-office workers, and the interval of the WGHE for office workers being extended from 1 year to 2 [26]. In 1995, when the national health examination service was transferred to the Ministry of Health and Welfare, the coverage of national health examination services was expanded to include the general population in accordance with the National Health Insurance Service Act. As the national NGHE was acknowledged as a type of general health examination service, the expenses for the WGHE, which had previously been borne by business owners, were taken up by the National Health Insurance Service. The enforcement regulations of the Industrial Safety and Health Act were again revised in 1997, thus leading to the abolishment of business owners’ obligation to report general health examination results. In 2002, every workplace with 1 or more workers was required to offer health examination services, while 2005 saw the discontinuation of health examinations specifically targeting new employees. Since then, the NGHE for workplace policyholders has replaced the WGHE according to the Industrial Safety and Health Act [24].

### The increase in the WGHE participation rate and health effect

We found that participation rates have rapidly increased over the last decade, from 51.6% in 2005 to 56% in 2006 and over 70% in 2011; since then, the rates have continued at a steady rate of around 70%. It is possible that these findings are the results of increasing income, the low-cost medical services of the NGHE, and a chronic disease prevention project. Another possibility is that the Industrial Safety and Health Act's mandating business owners to provide general health examinations for workplace policyholders has helped maintain the relatively high participation rates among such policyholders. Indeed, the participation rate of workplace policyholders was already 77% in 2006, which was substantially higher than the participation rate of the total population of the NHIS beneficiaries. Since 2007, the rates have been continually high, reaching upwards 80%. Another reason for the high participation could be the fact that examination agencies dispatch examiners to each workplace to provide these services, making them easily accessible by workers. However, it should be noted that a number of problems with the onsite health examination service have been pointed out, and its discontinuation has been discussed [1]. Thus, the effectiveness of this service should be discussed further in the future.

Regarding the comparison of participation rates among different enterprise sizes, we noted that the participation rates were lower among workers employed at enterprises with fewer than 50 employees than among those with 50 or more employees, and this gap remained consistent over the study period from 2006 to 2013. A possible reason for this is that National Health Insurance Program began by targeting workers of relatively large enterprises, who are more likely to be able afford to pay health insurance taxes; it has only rather recently expanded to workers from smaller enterprises and the self-employed, who generally have more unstable incomes. However, the consistent gap in health examination participation may mean that there remains a disparity in health examination opportunity due to socioeconomic status. In summary, participation rates are high overall, but there remain problems of unequal opportunities of health examination [27].

As for the age groups, the participation rates among the older generations—those in their 40s and 50s—were relatively low from 2006 to 2013 compared to examinees in their 20s and 30s, although the rates of each age group have been increasing. It should be noted, however, that these are merely the participation rates of NGHE beneficiaries. The dependents and household members of individuals in their 20s and 30s who were not workplace or regional policyholders would not be included among such beneficiaries. According to the Law for Health Promotion in the Republic of Korea [7, 28], only dependents or household members of an employee subscriber (i.e., workplace policyholder) or district subscriber (i.e., regional policyholder) who are 40 years old and above can receive the NGHE every other year. Thus, the participation rates of individuals in their 40s and above may reflect the total rate of all of these individuals in the NGHE because they are all given the opportunity to take it for free. In contrast, the dependents of national health subscribers below 40 years old would be excluded from the opportunity to take the NGHE. Thus, our results do not indicate that certain age groups show higher participation rates, but rather illustrate the number of beneficiaries in each age group who actually took the health examination.

### Follow-up management of health examination and secondary examination participation rates

In general, there is no common formal definition of concepts such as OHEs, periodic health exams, and screenings. Nevertheless, they all refer to health screening services utilizing tests to identify possible disease [13]. Additionally, the basic purpose of such examinations is to prevent targeted diseases and promote health. To this end, routine screenings and immunizations are strategically performed [29].

Article 52 of the National Health Insurance Service states that the “The Corporation shall conduct health examinations to find diseases among policyholders and their dependents as early as possible and to provide medical care benefits.”[28] Furthermore, Article 43 of the Industrial Safety and Health Act and Article 98 of the enforcement regulation of that same act state that “health examination shall be conducted regularly for workers’ health management”[30]. In other words, it may be considered that the concept of health examination includes efforts for improving the health conditions of examinees with abnormal results in addition to providing diagnoses.

Despite the increasing participation rates for the primary health examination, we observed no concomitant increase in the secondary health examination for examinees with abnormal results to ensure early detection and treatment. As was suggested in the secondary health examination results, we assume that this is due to the lack of follow up management in the NGHE system. Follow-up management refers to “additional intervention for those who are found to need further measures as a result of screening such as confirmation of a diagnosis, education, and consultation” [1]. In other words, the combination of a health examination with follow-up management would include all the procedures necessary to designate which examinees with abnormal results should visit medical centers for treatment or management services. Problems in this regard have been pointed out in the past as well [22, 31]. Furthermore, the ratio of examinees with abnormal results of diabetes and hypertension who visited outpatient department hospitals to diagnose their abnormal results and receive a prescription was low. This accords with a previous study among examinees who received medical treatment—specifically, those who had visited a medical center for their hypertension or diabetes with 90 days of examination accounted for only 2.21% and 1.18%, respectively [32].

Notably, the secondary examination participation rates were substantially higher among workplace policyholders than among other subjects; however, their participation rates did not show any increases over the study period. In particular, in comparing secondary examination participation rates among the different enterprise sizes, we found that participation rates in small enterprises (those with less than 50 employees) decreased somewhat over time, and were far below the rates of workers in enterprises with 50 or more employees. This gap in participation rates was even greater for the secondary examination than for the primary. Previous studies have shown that, among examinees who were suspected of having diabetes or showed abnormal results on the fasting glucose level test, only 5.66% sought out further medical examination for diabetes within 90 days [33]. In addition to follow-up management, there should be further research on the contribution of health examinations—both secondary and primary—to early diagnosis and treatment.

It may be helpful for specialized agencies for workplace health management and their affiliated health care professionals to fulfill the health management tasks of small-to-medium size enterprises through utilization of the current system. According to one survey conducted among medical practitioners of specialized agencies for workplace health management, workers showed a high level of compliance with advice for further medical treatment when diagnosed with abnormal results as a result of a health examination [34]. Thus, making good use of the existing system will be a way of maximizing the preventive effects of health examinations.

### Prevalence of diabetes and hypertension

**Fig. 1:**
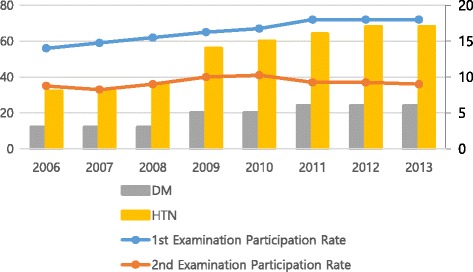
Changes in participation rates and the prevalence of diabetes and hypertension. Note: DM, diabetes; HTN, hypertension

Our results indicated that the prevalence rates of diabetes and hypertension have increased since 2006. This coincides with the increase in participation rates during that same period. Similarly, the prevalence rates leveled off at the same point—around 2011—as did the participation rates. However, these findings merely indicate the similarities in the graphs of participation rates and prevalence rates of diabetes and hypertension; they do not prove that higher participation rates indicate a higher probability of detecting more patients with diabetes and hypertension. In order to control for various factors possibly influencing this similarity, such as aging of the screening population, in the future, it would be necessary to observe the situation over the long term (Fig. 1).

As noted above, high blood glucose and blood pressure can be risk factors of CCVDs. Diseases of the circulatory system are the second most common cause of morbidity and mortality next to cancer in the Republic of Korea. Furthermore, CCVDs are compensable occupational diseases in the Republic of Korea, as in Japan or Taiwan [35]. They are a major cause of death and workforce loss among workers as well as in the general population in the Republic of Korea. When we classified workplace policyholders by their enterprise size, we noted that the prevalence and odds of having abnormal results relating to diabetes and hypertension were relatively higher among workers at an enterprise with less than 50 employees, who also demonstrated low participation rates. This same trend was found among the different age groups as well. As was mentioned above, the national health care and worker health care policies in Republic of Korea have only recently expanded their coverage from large enterprises to small ones [21]. Thus, the disparity between workers of these two types of companies indicates a health disparity due to socioeconomic status. Socioeconomic disparities in the prevalence of CCVDs and their risk factors in the Republic of Korea [36] and other countries [37, 38] have also been described in other studies. However, Korean examination agencies are allowed to dispatch their examiners to each workplace to provide such services. It is possible that examinees' blood pressure in the onsite examination is not as stable as that measured in the hospital, and some examinees, particularly those who have just a finished night shift, may not have fasted before a health examination.

### Health examination opportunity and health disparity

Small enterprise workers have less WGHE opportunity and have more abnormal results relating to diabetes and hypertension. A reason for the lower participation rates and higher rates of diabetes and hypertension among workers from smaller enterprises may be related to inequality in health and socioeconomic conditions. Specifically, the Ministry of Employment [39], the difference in monthly wages increases along with the scale of the business entity in South Korea: namely, the monthly wage at business entities with 300 or more employees was about 147% that of entities with 10–29 employees. When the difference in monthly wages between enterprise size was examined according to the Korean standard statistical classification of occupation (http://kssc.kostat.go.kr/ksscNew_web/ekssc/main/main.do), service workers (170%) and craft and related trades workers (165%) showed the largest gaps in monthly wages, while managers (116%) showed a relatively smaller gap. These differences in wages, along with other socioeconomic factors, may help to limit opportunities for health examination. Further support for this comes from a previous cohort study, wherein the occurrence of cardiovascular diseases was observed among examinees for 7 years [27]. The authors found that the occurrence rate was lower among individuals who regularly underwent health examinations. Furthermore, the subjects were broken down and examined by insurance type—non-office workers, who could take a health examination every year; office workers and regional policyholders, who would take a health examination every two years; and dependents of these policyholders for comparison. The results revealed that the gap in occurrence rate of cardiovascular disease between individuals who took health examinations regularly and those who did not was smallest among non-office workers (who could take a health examination every year). Thus, relatively healthy workers with better working conditions appear to be given more opportunities for health examination. Taken together, these previous studies indicate that the gap of health conditions narrows as the interval between health examinations shortens, suggesting that health examinations can serve as a social safety net so long as they are conducted regularly and further necessary treatment is provided through follow-up management.

Cho et al. pointed out that the differing participation rates by socioeconomic factors is one problem of the Korean national health examination system [40]; Myeong et al. also noted a participation disparity of workers in small workplaces (i.e., those with less than 50 employees): they estimated the odds ratios of participation in the WGHE by the size of enterprise, and found that workers employed at enterprises with less than 50 workers were less likely to participate in the WGHE than were those employed at enterprises with more than 300 workers [41]. Among public officials, whose working conditions are relatively stable, the difference in participation rates by enterprise size was non-significant. Overall, our results suggest that the poorer working conditions at small workplaces may limit opportunities for health examination.

## Conclusions

This study examined the current condition of the WGHE and the NGHE using national data. We identified the annual changes in participation rates of the NGHE. We also analyzed the data in terms of occupational medicine. While the overall participation rate is increasing, we found that participation was lower among smaller enterprise employees.

Notably, this study has some limitations: First, among the many chronic diseases assessed in the examination, we examined only the prevalence and odds of diabetes and hypertension. However, the targeted diseases or subjects that can be assessed by the items of the current health examination are somewhat vague. It would be necessary for future research to target other diseases for specific organs, such as hematologic, liver, and nephrotic disease; urogenital disorders; and auditory disorder [31, 42], in order to grasp the current condition of workers’ health examination.

The WGHE can be regarded as a method of health surveillance for workers exposed to vocational dangers. Berlin et al. [43] defines health surveillance as “regular clinical and physiological examination conducted to protect health of workers exposed to hazardous substances and to prevent diseases.” To make the government and business entities adopt consistent policies regarding the promotion of vocational health and thereby improve working conditions, the International Labor Organization has presented Occupational Health Services Convention (C161) [6], which specifies 11 basic duties for the protection of workers’ physical and mental health and the necessary aspects of health surveillance to that end. It is noteworthy that the tests in the WGHE target specific organs rather than diseases, as this allows for the inclusion of the preventive effects of little-known diseases. In consideration of the fact that some production line workers may be exposed to potentially hazardous substances even if these substances are not specified by law, test items targeting uncertain diseases also can play a role in surveillance of potential dangers. Furthermore, prevention of cardiovascular diseases is one of the most important objectives of the WGHE and NGHE.

Second, the results regarding the prevalence and odds of diabetes and hypertension provide estimates of who may be in need of medical treatment, but cannot provide clear diagnoses. More specifically, the figures determined cannot be considered wholly accurate because of the following factors: not all health examinations were performed in a fasting state; the examination process was relatively quick and conducted by a large-scale workforce; poor examination conditions in the case of onsite health examination services; white coat syndrome among examinees, etc.

Third, the distinction between office workers and non-office workers might cause confusion. According to domestic health examination law, office work is limited to clerical workers separated from production sites and do not engage in interactive services. Since job concepts in modern society are highly diverse, the concept stated in this study may be different from the concepts among manual workers/non-manual workers or blue-/white-collar workers. Furthermore, many of those who are classified as non-office workers primarily work at an office, whereas individuals that can be classified as white-collar according to commonly accepted notions may be classified as non-office workers.

Finally, we did not adjust for population age. As the population age structure changes annually, not controlling for it precludes detailed comparison and application of the results to specific groups. Nevertheless, the national data include all national health insurance beneficiaries. We also compared the data annually so that the differences in the population structures of each year were not large enough to produce a severe confound.

Despite these limitations, our study is of significance in that it clarifies the differences between the primary and secondary examination participation rates and the differences in the occurrence of diabetes and hypertension by enterprise size. Our results regarding enterprise size suggest that many workers at small workplaces suffer from inequality of health conditions and opportunities for health examination; given that the majority of domestic workers are now working in enterprises with less than 50 employees [39], these results are particularly relevant today.

Our study also suggests the need to take note of realistic participation rates along with the medical effects when deciding on the appropriate interval between health examinations, as low participation rates can result in extension of the actual health examination interval. Furthermore, medical guidelines for health examination intervals may vary depending on the target population’s accessibility to health examinations. Notably, compared to past health examination services, participation in the current health examination service was relatively high. However, in terms of the prevention of diseases, one of the purposes of health examination, it will be helpful to consider long-term average participation rates rather than participation rates of a single health examination in determining an effective interval of examination. For health examination to contribute to the prevention of diseases, studies of both of the examination participation rates and follow-up management are necessary. Supplementary measures also need to be developed for groups found to be in poor condition.

## Abbreviations

NGHE: National general health examination
NHIS: National health insurance service
OHE: Organized health examination.
WGHE: Workers’ general health examination
WSHE: Workers’ special health examination
