# Erratum to: The current condition of the workers’ general health examination in South Korea: a retrospective study

**DOI:** 10.1186/s40557-017-0174-z

**Published:** 2017-06-20

**Authors:** Young Joong Kang, Jun-Pyo Myong, Huisu Eom, Bowha Choi, Jong Heon Park, Eun-A L Kim

**Affiliations:** 1 Occupational Safety and Health Research Institute, Korea Occupational Safety and Health Agency, Ulsan, Republic of Korea; 2 Department of Occupational and Environmental Medicine, Seoul St. Mary’s Hospital, College of Medicine, Catholic University of Korea, Seoul, Republic of Korea; 3 Big Data Steering Department, National Health Insurance Service, Wonju, Republic of Korea

## Erratum

After publication of the original article [1] the authors found that the following affiliations were incorrect at the time of publication:

1. Jun-Pyo Myong
  The current affiliation,’ Occupational Safety and Health Research Institute, Korea Occupational Safety and Health Agency’ is incorrect.
  The corrected affiliation should be:
**Department of Occupational and Environmental Medicine, Seoul St. Mary’s Hospital, College of Medicine, Catholic University of Korea, Seoul, Republic of Korea**
2. Jong Heon Park
  The current affiliation,’ Occupational Safety and Health Research Institute, Korea Occupational Safety and Health Agency’ is incorrect.
  The corrected affiliation should be:
**Big Data Steering Department, National Health Insurance Service, Wonju, Republic of Korea**
